# StreamChol: a web-based application for predicting cholestasis

**DOI:** 10.1186/s13321-024-00943-9

**Published:** 2025-01-21

**Authors:** Pablo Rodríguez-Belenguer, Emilio Soria-Olivas, Manuel Pastor

**Affiliations:** 1 Research Programme On Biomedical Informatics (GRIB), Department of Medicine and Life Sciences, Universitat Pompeu Fabra, Hospital del Mar Medical Research Institute, Barcelona, Spain; 2 IDAL, Intelligent Data Analysis Laboratory, ETSE, Universitat de València, Valencia, Spain

**Keywords:** Framework, Web interfaces, QSAR, In-silico toxicology

## Abstract

This article introduces StreamChol, a software for developing and applying mechanistic models to predict cholestasis. StreamChol is a Streamlit application, usable as a desktop application or web-accessible software when installed on a server using a docker container.

StreamChol allows a seamless integration of pharmacokinetic analyses with Machine Learning models. This integration not only enables cholestasis prediction but also opens avenues for predicting other toxicological endpoints requiring similar integrations. StreamChol's Docker containerization also streamlines deployment across diverse environments, addressing potential compatibility issues. StreamChol is distributed as open-source under GNU GPL v3, reflecting our commitment to open science. Through StreamChol, researchers are offered a potent tool for predictive modelling in toxicology, harnessing its strengths within an intuitive and user-friendly interface, without the need for any programming knowledge.

**Scientific contribution ** This work offers a user-friendly web-based tool for cholestasis prediction and a complete workflow for creating web platforms that require the combination of both programming languages, R and Python.

## Introduction

In recent years there has been a noticeable shift towards embracing the 3 R’s principle—Reduce, Refine, and Replace—in animal testing, and to adopt New Approach Methodologies (NAMs) [1]. Among these methodologies, in silico toxicology (IST) methods are very attractive because of their cost-effectiveness and ethical considerations [2]. IST encompass a variety of approaches, including Quantitative Structure–Activity Relationship (QSAR) for hazard prediction, read-across for grouping or analogue identification, structural alerts creation, and docking simulations for predicting binding energies [3].

In this scenario, many researchers are actively producing valuable in silico tools with a high potential to be applied in practice. However, their use is often hampered by difficulties in accessing to the source code, intellectual property rights of some of the software components or practical difficulties in implementing them locally.

The implementation of IST tools as web applications can help to address some of the previously mentioned issues, as they offer a more intuitive experience of use, compared to command-line interfaces, making them more accessible [4]. Currently, there are numerous frameworks for web application development, each with its advantages [5], even if some of them have a steep learning curve and require that the developers have good skills as full-stack developers, being familiar with web-server (flask, Django, web3py) and frontend (React, Angular) programming languages. Streamlit (https://streamlit.io/) has been gaining popularity in recent years as a simpler, more accessible alternative since it was specifically designed for data scientists and machine learning (ML) engineers. In general, it provides a streamlined and user-friendly experience for building and deploying data applications, integrating seamlessly with popular data science tools and libraries. It was a popular choice for data-driven projects. For instance, Nápoles-Duarte et al. [6] developed an application for creating interactive molecular visualizations, enhancing the user experience with dynamic and engaging molecular displays. Lee et al. [7] developed StarGazer, which is a hybrid intelligence platform for drug target prioritization and digital drug repositioning. Another interesting streamlit application, tailored for drug discovery was created by Łapińska et al. [8] It employs ML and data integration to forecast drug impacts on serotonin, aiding in new drug identification and repurposing. Despite its simplicity and popularity, Streamlit may not be suitable for complex web development projects requiring advanced features or customization.

In this work, we describe a web-based application for developing and applying mechanistic-based cholestasis prediction models. The original models described in Rodríguez-Belenguer et al. [9] involve extrapolating in vitro inhibitory concentrations for multiple hepatic transporters to in vivo doses using in vitro to in vivo extrapolation (QIVIVE) methods [10] and comparing them with maximum therapeutic doses or with the expected dose in cases where the maximum therapeutic dose is not available. The application can be easily deployed in most computational platforms and used with no programming language installations.

## Implementation

### Overview

**Fig. 1:**
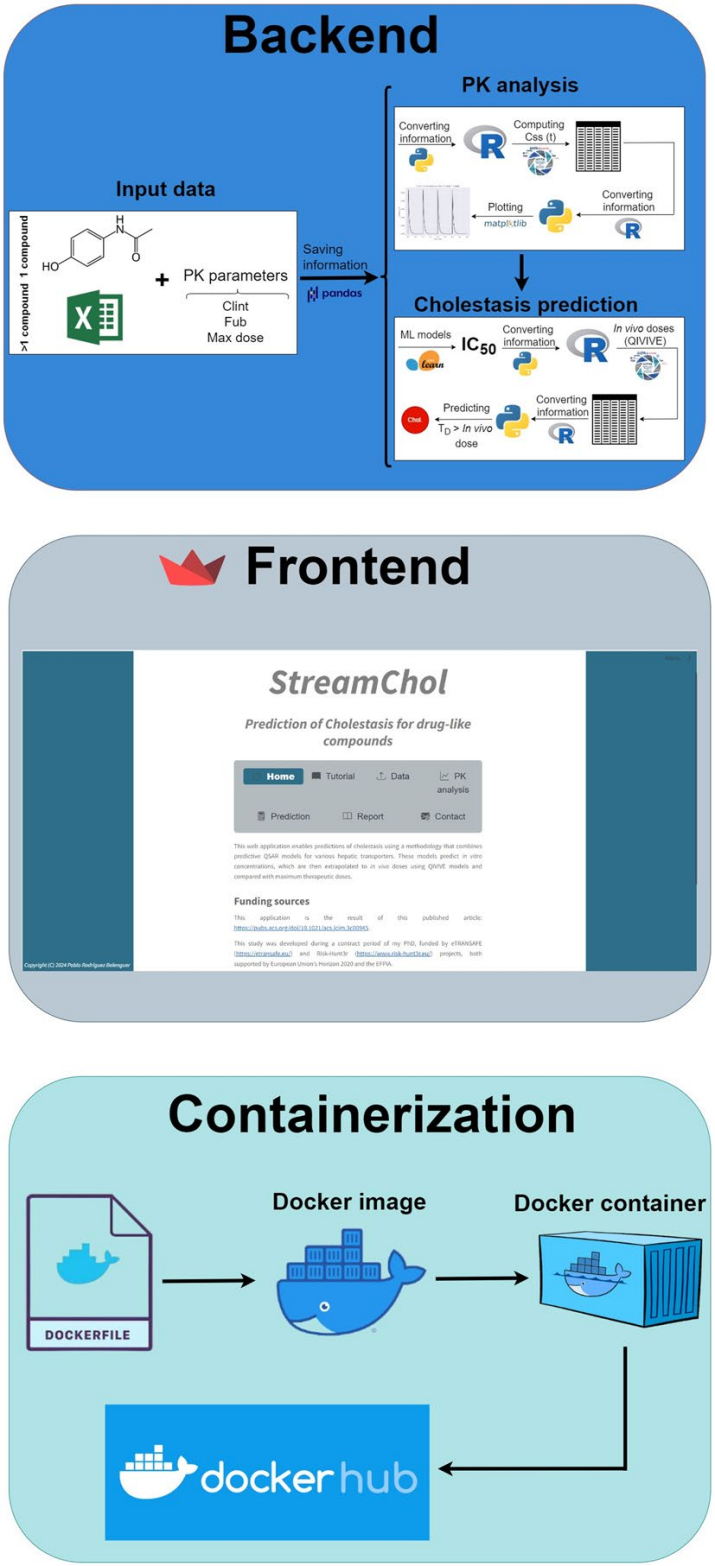
Schematic representation of the methodology for deploying a web application to predict cholestasis. The process is divided into three parts: backend, frontend, and containerization using Docker

StreamChol is a web application built on Streamlit, containerized using Docker, and distributed to the public through Docker Hub for predicting cholestasis (Fig. 1). The original model [9] for predicting cholestasis uses a mechanistic approach for cholestasis prediction. The model development and its use for prediction entails utilizing libraries from both R (for Pharmacokinetic [PK] analysis) and Python (for ML analysis), making the integration of R code with Python complex. Additionally, the combination of PK analysis with ML further increases the complexity of the model application.

Regarding the deployment of the application, although Streamlit provides the opportunity to deploy applications on its free cloud service, dependency issues can occasionally arise. Containerization offers a practical solution to this problem by provining a consistent application execution and dependency management across different systems, regardless of the underlying operating system. Docker [11] stands out as a widely-used platform for container creation, facilitating distribution through Docker Hub [12], a cloud-based registry that allows developers to store, share, and distribute Docker images.

### Main functionalities of StreamChol

The application's main screen shows tabs such as Home, Tutorial, Data, PK analysis, Prediction, Report, and Contact (see frontend block of Fig. 1). StreamChol has been designed to predict cholestatic activity for a single compound or multiple compounds (Data tab). In the first case, the compound structure can be entered using SMILES notation or drawn using a Sketcher tool, along with some descriptive (name, ChEMBL ID), pharmacokinetic (unbound fraction (FUB), intrinsic clearance (CLint)), and exposure (max dose) data, along with the target variable (Activity). In the second case, the same variables must be entered in an Excel file, with columns containing the following information (in exact order): name, ID, SMILES, Doses max, Activity, FUB, and CLint.

Once the structures to be processed are entered, StreamChol offers to evaluate plasma concentration–time curves at steady state (Css) along with Css curves at different fixed daily doses in the PK Analysis tab. This analysis is crucial, as it enables a comprehensive understanding of a drug's behaviour within the organism over time through Css. Such insights serve as a fundamental step in deriving in vivo doses, underscoring the nuanced relationship between drug kinetics and physiological responses. To conduct these assessments, a physiologically based toxicokinetic (PBTK) model accessible via the httk library [13] is employed, containing distinct tissue compartments for the gut, liver, lungs, arteries, veins, and kidneys. To perform these analyses, it is necessary to input a series of parameters into the model, such as daily dose, dose per day, and the number of days of administration for each drug. After this selection, the application displays, along with the different graphs, a dropdown tab to interpret these graphs.

Finally, cholestasis is predicted in the Prediction tab, where the mechanistic approach proposed by Rodríguez-Belenguer et al. [9] is implemented. To conduct this analysis, we use estimators from eight individual QSAR models employing Random Forest, XGBoost, and Support Vector Machines machine learning algorithms (Table 1). These models were tailored for different hepatic transporters as considered in Rodríguez-Belenguer et al. [9] to predict the IC_50_ of the drug(s) under analysis.

**Table 1:** ML models for each hepatic transporter

Transporter	ML model
BCRP	Random forest [14]
MRP2	Random forest
MRP3	Support vector machines [15]
MRP4	Random forest
OATP1B1	Random forest
OATP1B3	Support vector machines
BSEP	XGBoost [16]
P-gp	Random forest

Then, the predicted IC_50_ values are extrapolated to in vivo doses through the httk tool and compared with therapeutic doses (T_D_). In this section, a correction factor on the in vivo doses (K) must be specified, along with the hepatic transporters to be considered for the analysis and the logical rule to combine them (OR, AND, Majority). If the OR rule is selected, a compound is predicted as cholestatic if the therapeutic dose (T_D_) is higher than the in vivo dose for at least one of the selected transporters. In the case of the AND rule, the T_D_ must be higher than the in vivo doses of all the transporters to predict a compound as cholestatic. Lastly, in the case of the Majority rule, a compound is predicted as cholestatic if the T_D_ is higher than the in vivo dose of more than half of the selected transporters.

StreamChol produces individual prediction if only one compound has been entered, or multiple predictions if information for multiple compounds has been entered through an Excel file. In both cases, users are provided with additional information on the calculated in vivo doses. For a single prediction, the result is displayed in green with a checkmark icon if it agrees with the experimental value, and in red with a cross icon otherwise. However, If predicting the activity of more than one compound, there is an option to download a CSV file with all the predicted information. Additionally, in both cases, results from a reference dataset (training series) of 426 compounds obtained from Rodríguez-Belenguer et al. [9] are provided, including various metrics such as sensitivity, specificity, Area Under the Curve (AUC) score, Matthew’s correlation coefficient (MCC), and accuracy.

### StreamChol architecture

The StreamChol interface has been built using an enhanced version of Streamlit called streamlit_option_menu (https://github.com/victoryhb/streamlit-option-menu), which allows for the creation of applications with more intuitive menus than a standard multipage app. This component lets users select a single item from a list of options in a menu, similar to st.selectbox(), but with a static list display instead of a dropdown. It supports configurable icons for each option and the menu title, and allows customization of CSS styles for most HTML elements in the menu.

What sets StreamChol apart from other prediction tools is its combination of pharmacokinetic analysis with machine learning, rather than using direct QSAR models based solely on structural information. However, this requires the integration of both R and Python libraries. However, Streamlit was designed to create web applications with a Python backend. To integrate these analyses into our application, we have used the rpy2 library (https://github.com/rpy2/rpy2), which allows to integrate both programming languages.

The input data entered into StreamChol is stored in the session_state through a specific button, along with any information that needs to be reused in other tabs. This ensures that each time the user interacts with the application, it does not have to perform calculations from scratch. Once input data are stored, it is necessary to navigate to the PK analysis tab to perform some pharmacokinetic calculations using the httk library, which is built in R. Therefore, Python objects such as integers, strings, or dataframes need to be converted to R objects before being used in R scripts. For example, in cases where a numerical value such as the daily dose, dose per day, or days of drug administration can be selected by the user, these objects can be converted from Python to R using the code provided in the streamchol.py file found within the GitHub repository: https://github.com/phi-grib/StreamChol. For strings, such as drug names, they are assigned using the r.assign function. Regarding dataframes, they are converted from pandas [17] dataframes to R dataframes using the py2rpy_pandasdataframe() function presented in the streamchol.py file. Once Python objects have been converted to R objects, they can be used in R scripts to generate pharmacokinetic analyses. All the information obtained in R is stored in dataframes and converted back to pandas dataframes, allowing it to be represented graphically using the functionalities of the matplotlib [18] library. When analyzing more than a single compound, the information is stored in the session_state as a list of dataframes. Two buttons with back and forward items can be used to navigate the list, visualizing each pharmacokinetic result.

When the “Cholestasis prediction” button is pressed in the Prediction tab, the application reads the estimator for each transporter. Subsequently, it predicts the IC_50_ value(s) of the compound(s) we have entered in the application. At the same time, it reads the PK parameters that we have previously saved in the application (daily dose, dose per day, and days) and applies them to the PBTK model, which forms the basis of the QIVIVE model, to extrapolate in vitro concentrations to in vivo doses. At this point, the same process as in the previous tab is applied (Python object—> R object—> R script—> Python object). So, the mechanistic model is built as a scikit-learn [19] estimator, enabling the utilization of all its functionalities. Based on the application's structure, if any of the hyperparameters are modified, such as the transporter to be considered, the process will repeat once the button is pressed again.

### Installation

The application can be run locally or via Docker. To run it locally, you need to download the content from the GitHub repository: https://github.com/phi-grib/StreamChol. Before executing the command “streamlit run streamchol.py”, you must install Python on your system and create a Python environment according to the requirements.txt file. Additionally, R must be installed along with the httk and dplyr libraries. Also, it will need to configure the environment where R is hosted in the streamchol.py file. In our case, it would be: os.environ[“R_HOME”] = ”C:/Program Files/R/R-4.3.1”.

To access StreamChol via Docker, a fully configured container can be downloaded from DockerHub and installed typing ‘docker run -d -p 8501:8501 parodbe/streamchol_app’ in a console. Then, the StreamChol will be accessible from any web browser at the address (http://localhost:8501).

## Results

**Fig. 2:**
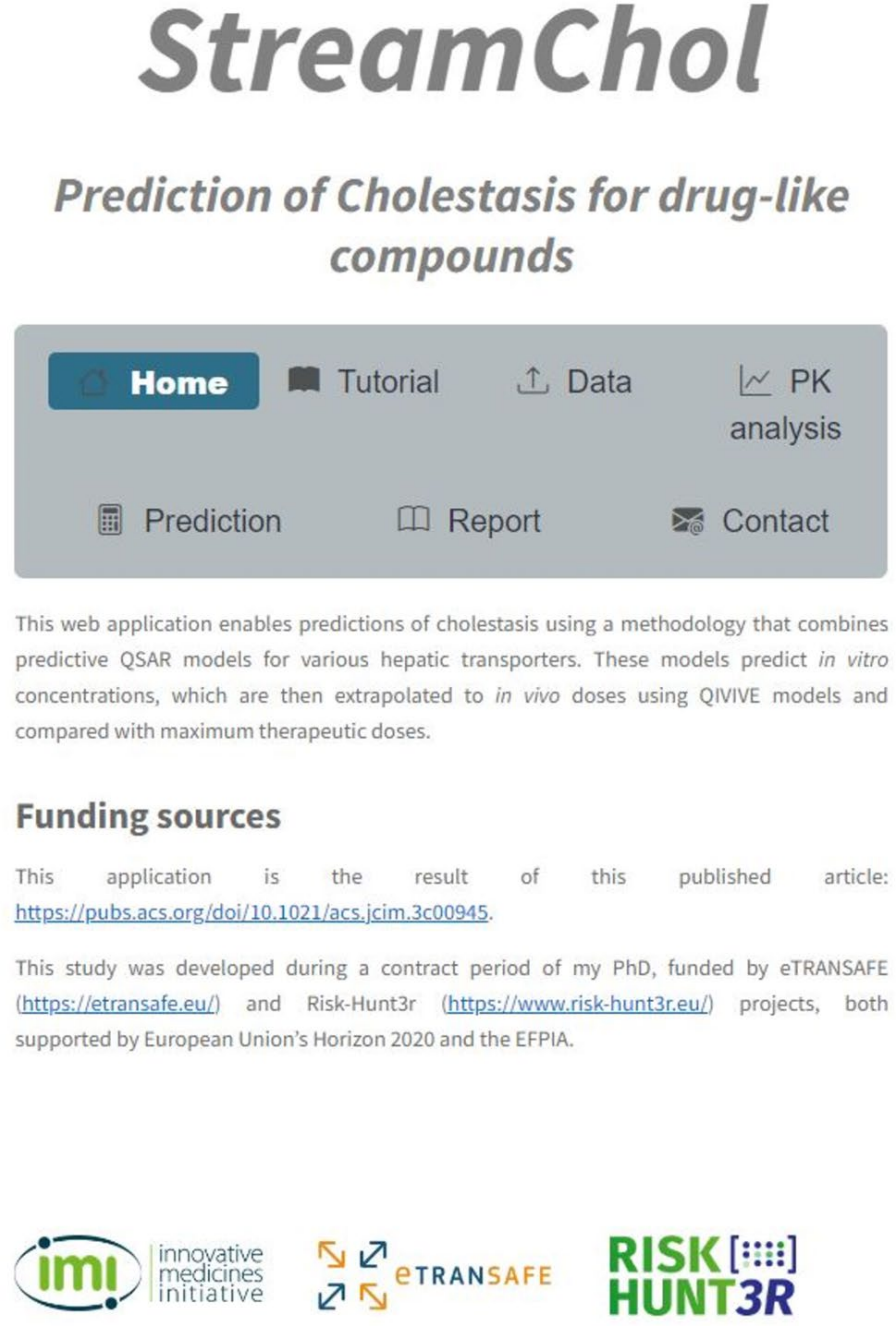
The user interface of the StreamChol application

The application Graphic User Interface (GUI) (Fig. 2) shows a layout of visual elements designed to provide a smooth and hassle-free experience. To learn how to use the application the Tutorial tab presents a step-by-step explanation of how to use StreamChol.

**Fig. 3:**
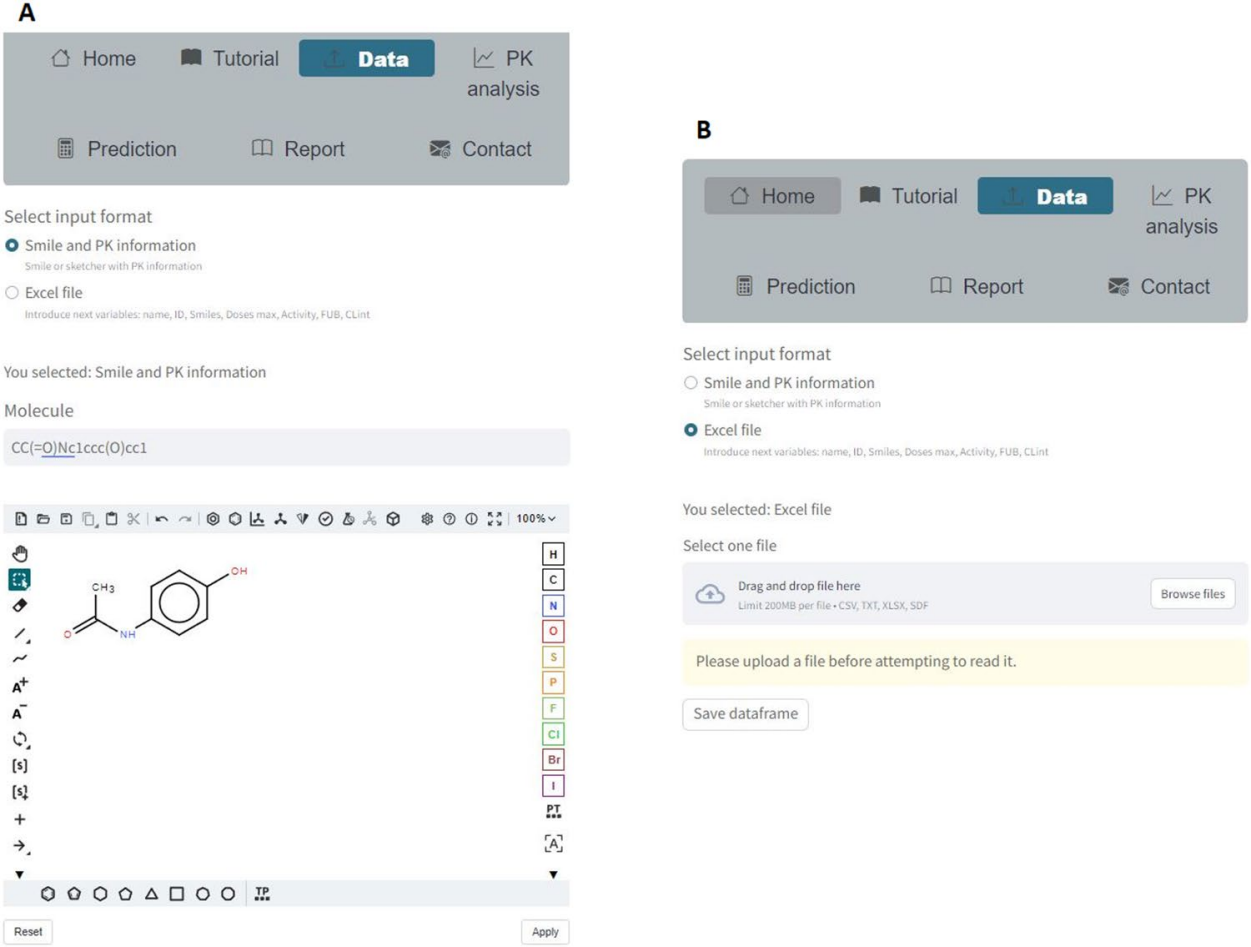
**A** Input data through Smile or Sketcher. **B** Input data through an Excel file

StreamCol allows to input information for a single drug-like compound, either entering its SMILE or by drawing the structure with the Sketcher, or for more than one compound by uploading an Excel file, as shown in Fig. 3. Afterward, the “Save Molecule” or “Save DataFrame” buttons are pressed in either cases to store the information in the session_state of the app for further analysis.

**Fig. 4:**
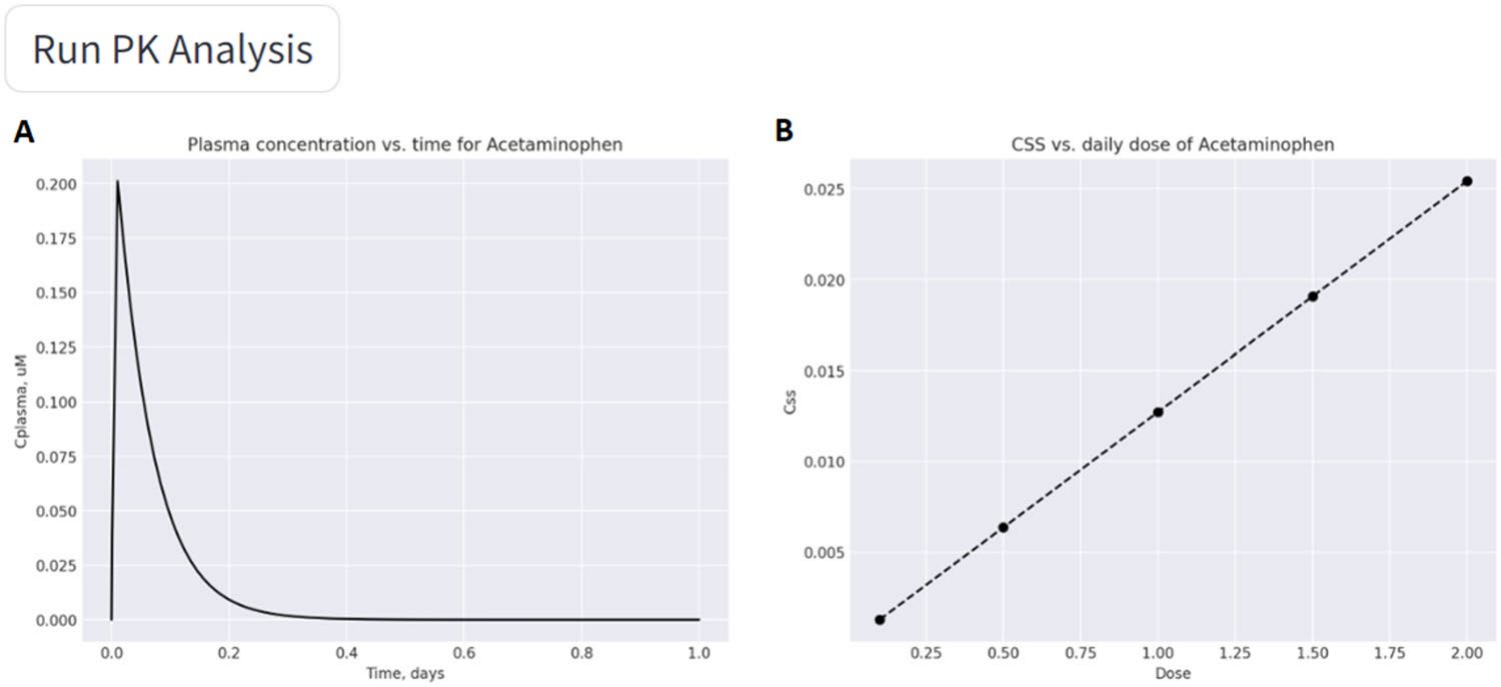
**A** Css against time. **B** Css againts dose

The next step is defining the administration days, daily dose, and the number of doses per day in the PK Analysis tab. Then StreamChol generates two graphs. In Fig. 4A, we can visualize the Css against time, and in Fig. 4B, the Css for different administered doses.

At the same time, a dropdown menu is provided with a brief interpretation of the generated graphs at a general level, which can help the user to interpret the obtained results.

**Fig. 5:**
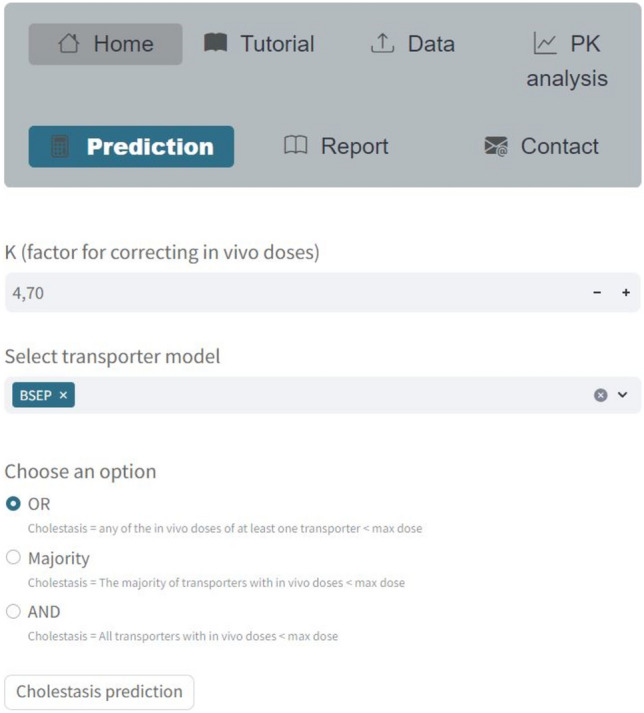
Screenshot of the Prediction tab

In the Prediction tab, It is necessary to specify the different hyperparameters of the mechanistic model mentioned above and press the “Cholestasis prediction” button in order to create individual transporter models for predicting IC_50_ and then extrapolate in vitro concentrations to in vivo doses (Fig. 5). Subsequently, these in vivo doses are compared with the maximum therapeutic doses based on the selected hyperparameters.

After building the model, the metrics mentioned above (Accuracy, Sensitivity, Specificity, MCC, and AUC) are provided for both the training series (426 compounds selected from the study published by Rodríguez-Belenguer et al. [9]) and the test series (the data entered via the Excel file in this case). These metrics will be computed only if the experimental values are available, which is not always the case in practice.

**Fig. 6:**
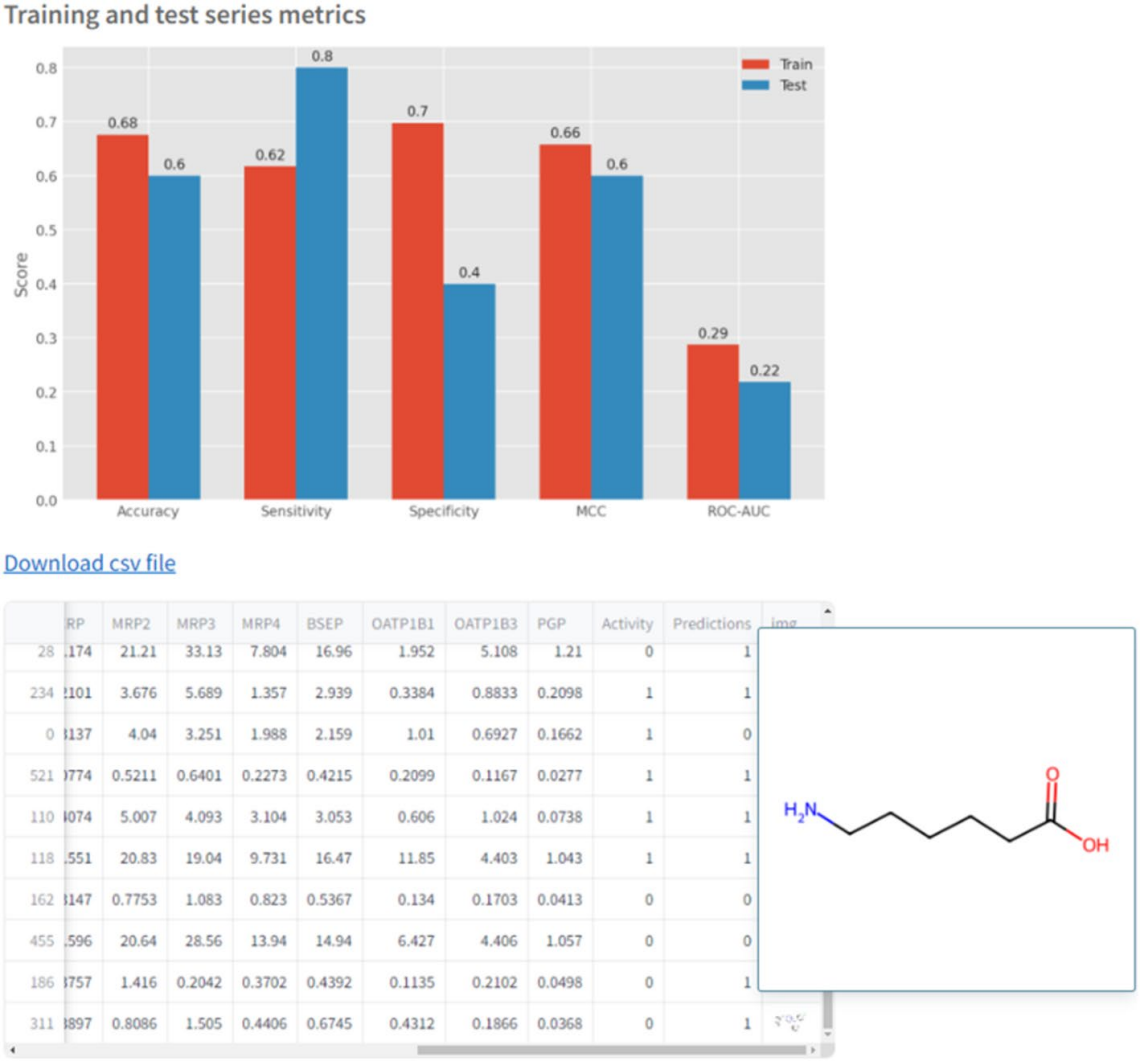
Screenshot with the results obtained in the Prediction tab

Additionally, a link has been enabled to download a CSV file containing the information we have entered along with the calculated in vivo doses and the predicted Activity. This CSV file be visualized in the application as a table with an additional column containing a representation of the compound structures which can be enlarged by double-clicking on the image (Fig. 6).

## Conclusion

We presented StreamChol, an open-source modelling framework for cholestasis prediction. It bridges the gap between complex scientific methodologies simplifying their application in practice. StreamChol has a user-friendly graphic interface for cholestasis prediction, facilitating quick and efficient access to predictions for researchers. By simplifying the process, StreamChol enables users to focus more on data interpretation and analysis, rather than the technical intricacies of model implementation, particularly in settings where time and resources are limited.

StreamChol addresses the inherent challenges associated with integrating code written in different programming languages. By integrating R and Python code within a single application, StreamChol simplifies the workflow for researchers, eliminating the need to navigate between different environments and ensuring seamless integration of diverse analytical techniques.

Additionally, one significant advantage of developing a web application that can be installed both locally and via a Docker image is that it allows companies to protect the privacy of their drug-like compounds. Local execution ensures that sensitive data remains within the company’s secure infrastructure, mitigating the risk of data breaches. In our case, the decision to containerize StreamChol using Docker provides the stability and scalability required, particularly in cloud-based deployment scenarios. Containerization offers numerous benefits, including improved reproducibility, as the application runs consistently across different environments; enhanced portability, as the Docker image can be easily shared and deployed; and resource isolation, which ensures that the application operates independently of the host system’s configuration. These features make Docker an ideal solution for deploying complex scientific applications in diverse computing environments, whether on local machines, private clouds, or public cloud platforms.

StreamChol predicts the cholestatic activity of compounds using input data such as compound name, ChEMBL ID, CLint, FUB, and maximum dose or activity. It evaluates Css curves over time and predicts cholestatic activity using the mechanistic model.

The challenges encountered during the development process underscore the importance of open science and collaboration within the research community. By openly sharing the code and methodologies used in StreamChol, researchers can benefit from collective expertise and contribute to ongoing efforts to advance computational toxicology and drug safety assessment.

Furthermore, StreamCol code is reusable and can serve as a template for other similar applications predicting different endpoints. By overcoming critical hurdles in model implementation, integration, and deployment, StreamChol enables researchers to leverage advanced scientific methodologies efficiently.

## Data Availability

All relevant code, pre-trained models, and datasets analysed in this study have been made publicly available on the GitHub repository referenced in the article: https://github.com/phi-grib/StreamChol.
